# Is Mate Preference Recognizable Based on Electroencephalogram Signals? Machine Learning Applied to Initial Romantic Attraction

**DOI:** 10.3389/fnins.2022.830820

**Published:** 2022-02-11

**Authors:** Guangjie Yuan, Wenguang He, Guangyuan Liu

**Affiliations:** ^1^College of Electronic and Information Engineering, Southwest University, Chongqing, China; ^2^Institute of Affective Computing and Information Processing, Southwest University, Chongqing, China; ^3^Chongqing Key Laboratory of Nonlinear Circuits and Intelligent Information Processing, Southwest University, Chongqing, China; ^4^College of Psychology, Qufu Normal University, Qufu, China; ^5^Key Laboratory of Cognition and Personality, Ministry of Education, Faculty of Psychology, Southwest University, Chongqing, China

**Keywords:** aesthetic preference, mate choice, physiological signals, frequency band, hemispheric asymmetries

## Abstract

Initial romantic attraction (IRA) refers to a series of positive reactions toward potential ideal partners based on individual preferences; its evolutionary value lies in facilitating mate selection. Although the EEG activities associated with IRA have been preliminarily understood; however, it remains unclear whether IRA can be recognized based on EEG activity. To clarify this, we simulated a dating platform similar to Tinder. Participants were asked to imagine that they were using the simulated dating platform to choose the ideal potential partner. Their brain electrical signals were recorded as they viewed photos of each potential partner and simultaneously assessed their initial romantic attraction in that potential partner through self-reported scale responses. Thereafter, the preprocessed EEG signals were decomposed into power-related features of different frequency bands using a wavelet transform approach. In addition to the power spectral features, feature extraction also accounted for the physiological parameters related to hemispheric asymmetries. Classification was performed by employing a random forest classifier, and the signals were divided into two categories: IRA engendered and IRA un-engendered. Based on the results of the 10-fold cross-validation, the best classification accuracy 85.2% (SD = 0.02) was achieved using feature vectors, mainly including the asymmetry features in alpha (8–13 Hz), beta (13–30 Hz), and theta (4–8 Hz) rhythms. The results of this study provide early evidence for EEG-based mate preference recognition and pave the way for the development of EEG-based romantic-matching systems.

## Introduction

Finding an ideal partner is a prerequisite for achieving high-quality romantic relationships. However, finding an ideal partner in real life can be extremely challenging (Spielmann et al., 2013; Joel et al., 2017). Because mate selection is not only a multivariate process involving the integration and trade-offs of multiple preferences but is also influenced by many factors, such as gender, culture, and personal experience (Buston and Emlen, 2003; Thomas et al., 2020). However, opportunities always coexist with these challenges. This is precisely because of the challenge of this task, which has created a huge economic market for matchmaking services (Joel et al., 2017). In this market, matchmaking agencies strive to provide customers with “tailored” romantic matching services and earn huge returns on this. The success of such a business model hinges on finding key features from appropriate signals that can effectively identify a user’s initial romantic interest toward a potential partner, as this largely determines the effectiveness of a matching service and consequently whether a user is willing to pay for it (Joel et al., 2017).

The current mainstream approach taken by matchmaking companies is that when users register for romantic matching services, they are required to fill in a series of questionnaires about their own characteristics and preferences based on their subjective feelings; these answers will then be fed into the matching algorithm as features to match suitable potential partners for users. Many matchmaking companies claim that effective romantic pairing can be achieved in this manner (Joel et al., 2017). However, Joel et al. (2017) demonstrated that it was impossible to predict initial romantic desire using any combination of traits and preferences reported prior to dating. In other words, effective romantic pairing cannot be achieved using this method. For matchmaking companies that take this as the core selling point, this conclusion is undoubtedly very destructive. However, from the perspective of psychology, this conclusion is undoubtedly reasonable, because the self-reported data are easily affected by subjective consciousness and the surrounding environment, which makes many characteristics of the input matching algorithm invalid, thereby invalidating the matching algorithm (Lin et al., 2010; Alarcao and Fonseca, 2019).

The essence of initial romantic attraction (IRA) is a series of positive responses to potential ideal partners based on individual preferences, including positive emotional responses (such as feelings of exhilaration and craving for emotional union) (Fisher, 1998; Fisher et al., 2002, 2005; Gerlach and Reinhard, 2018; Yuan and Liu, 2021; Yuan et al., 2021). An individual’s internal emotional reaction can be revealed not only through subjective self-reports but also through internal expression (i.e., physiological signals) (Gunes et al., 2011; Alarcao and Fonseca, 2019). Moreover, physiological signals have many advantages over self-reported data, one of which is that they are less susceptible to subjective consciousness and environmental factors (Lin et al., 2010; Alarcao and Fonseca, 2019). Thus, these signals open up new possibilities for identifying users’ emotional responses and preferences for potential partners. For instance, Zhang et al. (2021) successfully identified participants’ initial romantic interest to potential partners based on the features extracted from electrocardiogram signals, while Lu et al. (2020) successfully detected participants’ initial romantic desire to potential romantic partners based on the information extracted from photoplethysmogram signals. These results demonstrate that IRA, as an important part of human emotion, can be recognized on the basis of periphery physiological signals (Lu et al., 2020; Zhang et al., 2021).

In addition to periphery physiological signals, signals captured from the central nervous system, such as EEG, functional magnetic resonance imaging, or positron emission tomography, have also been proved to provide informative information for emotion recognition (Lin et al., 2010; Alarcao and Fonseca, 2019). Furthermore, among the many biosignals recorded over the brain, EEG is considered to a preferred method in studying the brain’s response to emotional stimuli due to its characters of high temporal resolution, non-invasive, inexpensive and convenient (Niemic, 2004; Alarcao and Fonseca, 2019). Therefore, in the field of neurophysiology, some studies have begun to investigate brain activities associated with IRA based on EEG signals. For instance, using event-related potential source analysis, Yuan et al. (2021) found that the arousal of IRA will significantly enhance the activation intensity of emotional processing-related areas, including the orbital frontal cortex and insula; attention control-related areas, including the frontal eye field and cingulate cortex; visual processing-related areas; and social evaluation-related areas, including the left dorsolateral prefrontal cortex. In another study, Yuan and Liu (2021) used time–frequency (TF) decomposition technology and found that processing of individual face preferences that triggered IRA was associated with a decrease in power in the alpha and lower beta bands over the lateral occipital complex and vertex areas; they hypothesized that changes in alpha and beta power may reflect cortical activation related to emotional stimulus significance (Schubring and Schupp, 2019, 2021). In addition, numerous neuropsychological studies have demonstrated that the asymmetry between the two hemispheres of the frequency band (FB) (especially the alpha and beta bands) was correlated with emotional activities and preferences (Balconi and Mazza, 2009; Liu et al., 2011; Hadjidimitriou and Hadjileontiadis, 2012; Huang et al., 2012; Jatupaiboon et al., 2013; Alarcao and Fonseca, 2019).

In the field of neuroeconomics, although EEG signals have not been used to identify users’ emotional responses and preferences toward potential partners, they have been widely used to identify users’ emotional responses and preferences to other stimuli (Aldayel et al., 2020a,b,c, 2021; Khurana et al., 2021; Naser and Saha, 2021; Zheng et al., 2021). Among previous studies, many researchers have used frequency bands (FBs) as features (Aldayel et al., 2020c,^2021^; Khurana et al., 2021; Naser and Saha, 2021; Zheng et al., 2021). For example, Chew et al. (2016) measured the preference of virtual three-dimensional shapes using band power as a feature for two preference categories and obtained accuracies of up to 80%. Aldayel et al. (2020b) measured the preference of consumer using frequency bands features as the feature for two preference categories and obtained accuracies of up to 93%. Meanwhile, several studies on preference also used hemispheric asymmetry scores (ASs) as input features (Aldayel et al., 2020a,b; Naser and Saha, 2021). For instance, Hadjidimitriou and Hadjileontiadis (2013) measured the preference of music using band power and hemispheric ASs as features for two preference categories using the k-nearest neighbors to obtain accuracies of up to 86.52%. Moon measured the preference of visual stimuli using band power and hemispheric ASs as features for four preference categories, achieving accuracies of up to 97.39% (Moon, 2013; Chew et al., 2016).

Although EEG signals have been widely used to identify users’ emotional responses and preferences to other stimuli, and EEG activities associated with IRA have also been preliminarily understood, whether users’ emotional responses and preferences toward potential partners can be identified on the basis of EEG signals remains unclear. To clarify this, we simulated a mate selection platform similar to Tinder. Participants were asked to imagine that they used the platform to select potentially desirable partners. Their EEG signals were recorded when they viewed and rated the photographs of each potential partner according to their preferences. Specifically, during the EEG recording task, the heterosexual participants were asked to rate photos of opposite-sex potential partners on two dimensions: an four-point IRA rating scale (based on the question “How much would you like to date this person?”; response: “not at all,” “a little,” “somewhat,” or “very much”) as well as a three-point zero-acquaintance rating scale (based on the question “Have you ever seen the person in the photo before?”; responses: “no,” “not sure,” or “yes”) (Yuan and Liu, 2021; Yuan et al., 2021). The IRA scale was used to assess the romantic interest of participants toward potential romantic partners, because the desire for emotional union with potential partner is one of the main characteristics of initial romantic attraction arousal. The zero acquaintance scale was used to ensure that participants were at the same level of familiarity with the stimulus material. Numerous studies have demonstrated that the random forest classifier (RFC) performs well in preference classification tasks based on EEG signals; therefore, in this study, the RFC was used to classify and detect the users’ IRA toward potential partners based on features obtained through TF analyses.

## Materials and Methods

Both the auxiliary experiment and the main experiment were approved by the Ethical Review Committee of Southwest University.

### Auxiliary Experiment

#### Participants

Sixty student volunteers participated in the auxiliary experiment (30 women and 30 men; age: 21.4 ± 2.6 years). All participants reported normal or corrected-to-normal visual acuity and had no history of psychiatric or neurological disorders, as confirmed *via* a screening interview.

#### Experimental Procedure

The induction rate of IRA has been reported to be quite low (only a few percent) (Zsok et al., 2017), the IRA induction rate should be increased to obtain enough data to train the model (Yuan and Liu, 2021; Yuan et al., 2021). Numerous studies have shown that physical attractiveness is a good predictor of a an individual’s popularity (i.e., probability of being selected by the opposite sex) with the opposite sex (Asendorpf et al., 2011; Olderbak et al., 2017; Gerlach and Reinhard, 2018; Yuan et al., 2021). Therefore, in this study, we planned to increase the average induction rate of IRA by increasing the proportion of stimuli with high physical attractiveness (Yuan and Liu, 2021; Yuan et al., 2021).

To achieve this goal, we first assessed the attractiveness level of each stimulus. To assess the attractiveness level, we first focused on downloading thousands of high-resolution personal portrait photographs from a high-definition copyright commercial photograph library (i.e., Hummingbird^1^) and standardized them (face and hair only; size, 839 × 1,080 pixels). To control the interference factors, we then selected 1,600 photographs from the standardized portrait photograph library; the criteria for screening the photographs were similar orientation and expression of the face and comparable background complexity. Thereafter, the physical attractiveness level of each face was assessed using a nine-point Likert scale. Notably, the male participants rated only female faces, while the female participants rated only male faces. We then calculated the average attractiveness level of each face by averaging the ratings of the same face from 30 participants of the opposite sex. Finally, according to the average attractiveness level, these faces were divided into three categories: high attractiveness [mean = 6.9, standard deviation (SD) = 0.33], medium attractiveness (mean = 5.2, SD = 0.25), and low attractiveness (mean = 3.9, SD = 0.31).

In the natural environment, the proportion of individuals with high, medium, and low attractiveness should conform to the normal distribution. However, in this study, we deliberately increased the proportion of individuals with high attractiveness, reduced the proportion of stimuli with low attractiveness, and adjusted the ratio of high, medium, and low attractiveness to 0.25:0.6:0.15 to increase the average induction rate of IRA. The number of times each participant would need to be exposed to different stimuli was determined to be between 300 and 400 after the comprehensive trade-offs of induction efficiency and participant burden. Ultimately, 360 photographs were selected as the stimulus material for the main experiment for each sex from among 800 photographs of women and 800 photographs of men (Yuan and Liu, 2021; Yuan et al., 2021).

### Main Experiment

#### Participants

Fifty student volunteers participated in the main experiment (all single; 25 women and 25 men; age: 21.2 ± 2.4 years). All participants reported normal or corrected-to-normal visual acuity and had no history of psychiatric or neurological disorders, as confirmed *via* a screening interview.

#### Experimental Procedure

**FIGURE 1:**
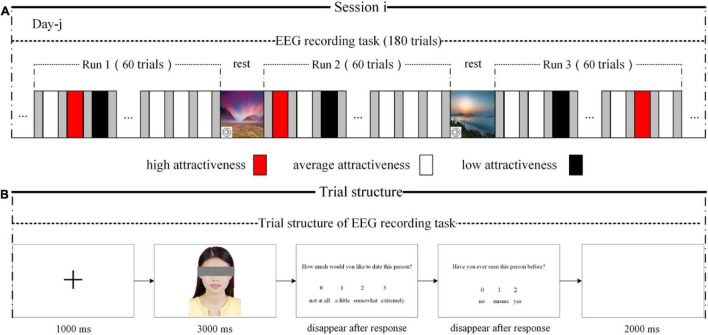
Experimental protocol and trial structure. **(A)** Experimental protocol. **(B)** Trial structure.

The number of stimuli used in the main experiment was significantly reduced by the aforementioned strategy; however, processing of 360 stimuli was still a high-load task for the participants. Specifically, when the participants were asked to complete the task over a short period, they were more likely to experience aesthetic fatigue, which may interfere with the experimental effect. Therefore, to minimize the probability of or delay aesthetic fatigue, we first divided 360 photographs of women (or men) equally into two sessions based on their attractiveness level and stipulated that the interval between completing the two parts of the experiment should be at least 1 day (Figure 1A). Thereafter, the 180 photographs from each session were divided equally into three runs using the same rules, and a 5–6-min break was provided between every two runs. During the rest period, the participants viewed serene landscapes while listening to soothing music. Notably, the experiment was conducted in a dark and quiet environment to keep the participants focused on the stimulus.

For each session, the trial structure of the EEG recording task is shown in Figure 1B. A black fixation cross appeared in the center of a white computer screen for 1,000 ms, followed by a photograph appearing for 3,000 ms. The participants were then asked to assess their romantic interest toward the potential partner based on the question, “How much would you like to date this person?” on a four-point rating scale (0 = not at all; 1 = little; 2 = somewhat; 4 = very much) (Finkel et al., 2007; Cooper et al., 2012; Gerlach and Reinhard, 2018; Yuan and Liu, 2021; Yuan et al., 2021). Thereafter, they were asked, “Have you ever seen the person in the photograph before?” (0 = no; 1 = not sure; 2 = yes). Finally, there was a 2,000-ms blank screen.

#### Data Acquisition and Processing

**FIGURE 2:**
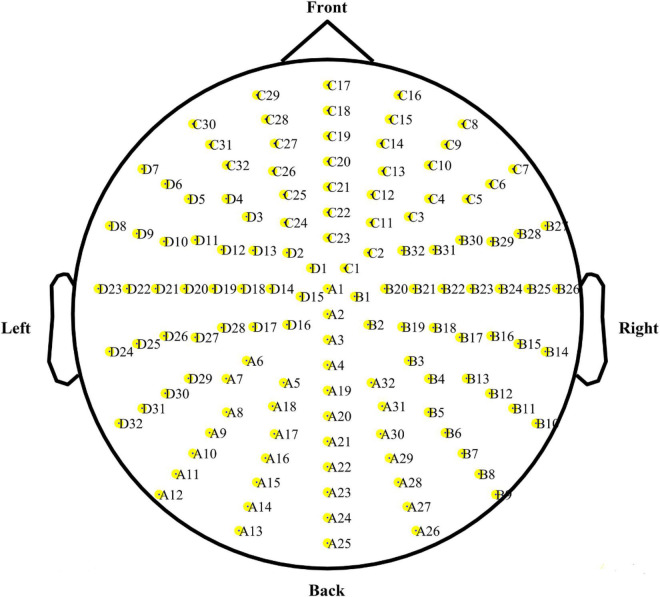
Electrode positions for the BioSemi activeTwo system.

The EEG signals were recorded using the 128-channel BioSemi ActiveTwo system (BioSemi Inc., Heerlen, Netherlands) with a 24-bit analog-to-digital conversion. The 128 electrodes were equally spaced on an electrode cap and customized with an integrated primary amplifier (Figure 2). The data were filtered online at a 0.16–100-Hz band-pass filter and sampled at 512 Hz (Yuan and Liu, 2021; Yuan et al., 2021). After the completion of data acquisition, the continuous EEG signals were re-referenced offline to the average of all channels after rejecting bad segments and interpolating bad traces; the bandpass filter ranged from 0.1 to 50 Hz. An independent component analysis was used to correct electrooculography artifacts from eye movements and blinks. The preprocessed EEG signals were split into epochs from 200 ms before the presentation of the stimulus to 2,000 ms after the onset of the stimulus. EEG data analysis was conducted using the open-source MATLAB signal processing toolbox FieldTrip and in-house functions in MATLAB (Oostenveld et al., 2011).

According to the score for “How much would you like to date this person?,” the EEG epochs were divided into IRA engendered and IRA un-engendered (Fisher et al., 2005; Gerlach and Reinhard, 2018; Yuan and Liu, 2021; Yuan et al., 2021). The IRA engendered category comprised the epochs in which the participants rated their IRA for the potential partners as 3 (very much) or 2 (somewhat). The IRA un-engendered category comprised the epochs in which the participants rated their IRA for the potential partners as 0 (not at all). To minimize ambiguity, we excluded epochs with a rating score of 1. The number of acceptable epochs under the IRA engendered category was 1439, while the number of all acceptable data segments in the IRA un-engendered category was 15298. To solve the problem of serious mismatch in the number of samples between the two preference categories, we randomly selected a number of accepted samples under the IRA un-engendered category to match the number of accepted samples under the IRA engendered category.

#### Feature Extraction

**FIGURE 3:**
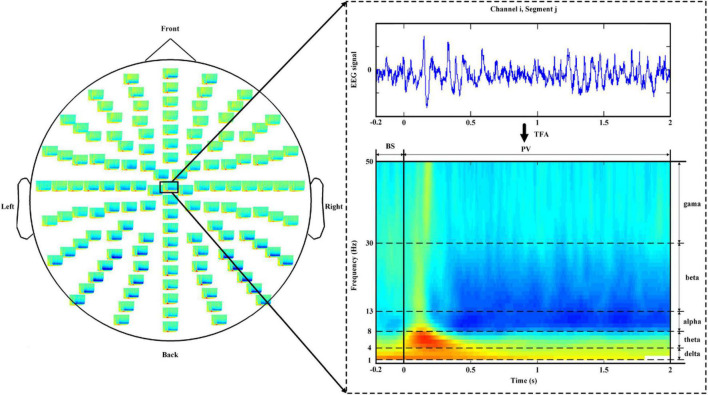
Time–frequency plane segmentation for the quantity estimation of B and V, from the TFP of the EEG signal corresponding to channel *i* and segment *j*. EEG, electroencephalogram. TFP, time–frequency representation.

To recognize the users’ discrete preferences, we used the wavelet transform (WT) with a sliding time-window approach for TF feature extraction based on the TF analysis (Lindsen et al., 2010; De Cesarei and Codispoti, 2011; Kang et al., 2015; Yuan and Liu, 2021). Specifically, the time–frequency representation (TFR) was obtained through a five-cycle complex Morlet WT. The sliding windows were advanced in 12-ms and 1-Hz increments to estimate the changes in power over time and frequency in the five FBs: delta (1–4 Hz), theta (4–8 Hz), alpha (8–13 Hz), beta (13–30 Hz), and gamma (30–49 Hz). The TF features of the EEG activities were calculated according to event-related oscillations (Pfurtscheller and Lopes da Silva, 1999; Hadjidimitriou and Hadjileontiadis, 2013; Liu et al., 2018; Yuan and Liu, 2021). In this study, two types of TF features were extracted: the power spectral feature (PSF) and the AS (i.e., difference in spectral power between the left and right hemispheres). For each epoch *j* and channel *i*, each PSF was computed as follows:


(1) $$\text{PSF}=\frac{\text{V}-\text{B}}{\text{B}}$$

where V represents the quantity estimated during the photograph viewing (PV) period, and B represents the quantity estimated during the baseline state (BS) period. To obtain the quantity V, we averaged the TFR during the PV period over the constituent frequencies and time (2). Similarly, B was computed in the same manner as in PV, as shown in (3).


(2) $${\text{V}}_{\text{w}}^{\text{fb}}\,\left(\text{i},\text{j}\right)=\frac{1}{{\text{N}}_{\text{w}}}\,\sum_{\text{t}}\left(\frac{1}{{\text{N}}_{\text{fb}}}\,\sum_{\text{f}}{\text{TFR}}_{\text{i},j}^{\text{PV}}\,\left[\text{t},f\right]\right)$$

(3) $${\text{B}}_{\text{w}}^{\text{fb}}\,\left(\text{i},\text{j}\right)=\frac{1}{{\text{N}}_{\text{w}}}\,\sum_{\text{t}}\left(\frac{1}{{\text{N}}_{\text{fb}}}\,\sum_{\text{f}}{\text{TFR}}_{\text{i},j}^{\text{BS}}\,\left[\text{t},f\right]\right)$$

where [t, f] represents the discrete (time and frequency) points in the TF plane; TFR*^PV^* represents the obtained TFR during the PV period (Figure 3); and N_*w*_, N_*fb*_ denote the number of sample points in the time window of 0–2 s and the number of frequency bins in each FB, respectively (Yuan and Liu, 2021). Similarly, TFR*^BS^* represents the obtained TFR during the BS period (Figure 3), and N_*w*_, N_*fb*_ denote the number of sample points in the time window of −0.2–0 s and the number of frequency bins in each FB, respectively. Herein, a TFR*^BS^* was used to correct the emotional baseline of the TFR*^PV^* to exclude the confounding effects of other factors.

In addition to the PSFs, the ASs of all 55 symmetrical pairs of electrodes on the left and right hemispheres in the five FBs were extracted to measure the possible lateralization of brain activity that might be caused by emotional stimuli (Liu et al., 2018; Yuan and Liu, 2021). In general, a total of 915 (640 PSFs and 275 ASs) EEG features were extracted.

#### Preference Recognition With Feature Selection

Nine hundred and fifteen features were extracted from the EEG signals on 128 electrodes, which is undoubtedly a high-dimensional dataset. To effectively analyze the data and save computational resources, we conducted necessary feature selection before classification (Lu et al., 2020; Zhang et al., 2021). The paired sample *t*-test was used to screen out the feature subsets with significant differences between the IRA engendered and IRA un-engendered categories. A total of 188 features with significant differences (*p* < 0.05) were identified. On this basis, the recursive feature elimination with cross-validation sequential forward feature selector (RFECV) was applied to conduct further feature selection.

**FIGURE 4:**
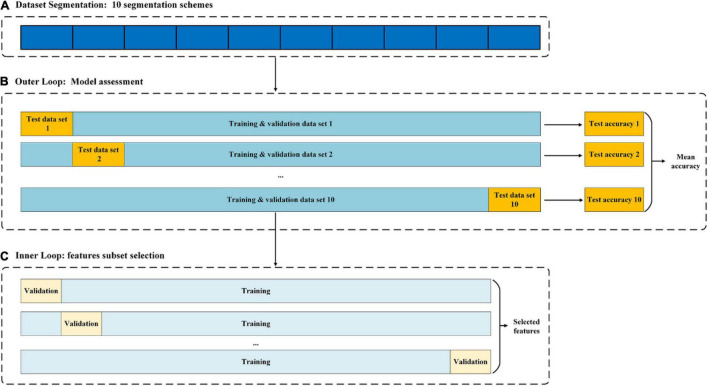
Nested cross-validation architecture used for feature selection and model assessment.

To use the entire dataset to train and test the classifier, we used a nested 10-fold cross-validations to obtain reliable model estimates for feature selection and model training (Pourmohammadi and Maleki, 2020; Zhang et al., 2021). Specifically, the inner loop was responsible for selecting the optimal subset of features (Figure 4). In the outer loop and using the selected subset of features, the RFC was evaluated by unseen test data set *via* a subject-wise 10-fold cross-validation (Saeb et al., 2017). Thereafter, the confusion matrix was formed based on the true and predicted labels of sample in the each unseen test data set. Then, based on the confusion matrix, common metrics are calculated to assess performance of machine learning system, including classification accuracy (CA), sensitivity (SE), specificity (SP), area under curve (AUC), Jaccard index (JI), F-measure (FM), and polygon area metric (PAM) (Aydemir, 2020). The mathematical definitions are, respectively, given as follows:


(4) $$C\,A=\frac{T\,P+T\,N}{T\,P+T\,N+F\,P+F\,N}$$

(5) $$S\,E=\frac{T\,P}{T\,P+F\,N}$$

(6) $$S\,P=\frac{T\,N}{T\,N+F\,P}$$

(7) $$J\,I=\frac{T\,P}{T\,P+F\,P+F\,N}$$

(8) $$F\,M=\frac{2\,T\,P}{2\,T\,P+F\,P+F\,N}$$

(9) $$A\,U\,C=\int_{0}^{1}f\,\left(x\right)\,d\,x$$

Where TP is the number of actual positive samples that were predicted to be positive, FN is the number of actual positive samples that were predicted to be negative, TN is the number of actual negative samples that were predicted to be negative, and FP is the number of actual negative samples that were predicted to be positive (Aydemir, 2020). The classifier selected in this study is a widely used classifier with good performance, namely, the RFC (Aldayel et al., 2020a,b). For the RFC, the Gini impurity was used as a function to measure the quality of a split; the maximum depth of the tree was set to 30; and the other super parameters were set to default.

## Results and Discussion

**FIGURE 5:**
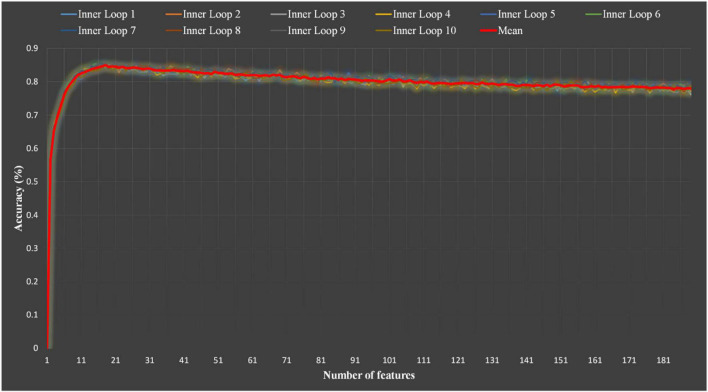
Classification accuracy using the RFC with different numbers of features. RFC, random forest classifier.

The classification performance of the proposed EEG-based mate preference recognition algorithm was verified using a total of 2878 EEG samples (including 1439 samples of the IRA engendered category and 1439 samples of the IRA un-engendered category) collected from 50 participants. To obtain an optimal feature subset from 188 features with significant differences (*p* < 0.05) between the two categories, we used a nested 10-fold cross-validation scheme based on the RFECV-RFC algorithm for feature selection. The number of features varied from 1 to 188, and the best feature subset was selected in each step. Figure 5 displays the mean classification accuracies on the validation sets of each inner loop when selecting different numbers of features. As can be seen from Figure 5, the number of features of the optimal feature subset selected by each internal cycle is roughly the same (about 17, SD = 0.57). The performance of the model is evaluated on the corresponding test set based on the optimal feature subset selected in each inner loop. The results are shown in Tables 1, 2. It can be seen from Table 2 that the best mean CA value, mean PAM value, mean se value, mean SP value, mean AUC value, mean Ji value and mean FM value are 0.8528, 0.6990, 0.8566, 0.8486, 0.8540, 0.7439, and 0.8530, respectively.

**TABLE 1:** Confusion matrix of each test data set.

	True label Predicted label	IRA engendered	IRA un-engendered
Test data set 1	IRA engendered	144	27
	IRA un-engendered	20	179
Test data set 2	IRA engendered	163	23
	IRA un-engendered	41	141
Test data set 3	IRA engendered	123	19
	IRA un-engendered	20	130
Test data set 4	IRA engendered	139	25
	IRA un-engendered	25	125
Test data set 5	IRA engendered	117	16
	IRA un-engendered	17	116
Test data set 6	IRA engendered	118	23
	IRA un-engendered	18	100
Test data set 7	IRA engendered	137	25
	IRA un-engendered	26	107
Test data set 8	IRA engendered	110	22
	IRA un-engendered	14	105
Test data set 9	IRA engendered	98	21
	IRA un-engendered	17	101
Test data set 10	IRA engendered	80	14
	IRA un-engendered	13	119

**TABLE 2:** The results of each test data set.

Metrics	PAM	CA	SE	SP	AUC	JI	FM
Test data set 1	0.72	0.8730	0.8780	0.8689	0.87	0.7539	0.8597
Test data set 2	0.66	0.8261	0.7990	0.8598	0.83	0.7181	0.8539
Test data set 3	0.72	0.8664	0.8601	0.8725	0.87	0.7593	0.8632
Test data set 4	0.68	0.8408	0.8476	0.8333	0.84	0.7354	0.8476
Test data set 5	0.74	0.8579	0.8731	0.8788	0.88	0.7800	0.8764
Test data set 6	0.68	0.8417	0.8676	0.8130	0.84	0.7241	0.8520
Test data set 7	0.74	0.8271	0.8405	0.8106	0.83	0.7287	0.8431
Test data set 8	0.71	0.8566	0.8871	0.8268	0.86	0.7534	0.8594
Test data set 9	0.67	0.8397	0.8522	0.8279	0.84	0.7206	0.8376
Test data set 10	0.73	0.8805	0.8602	0.8947	0.88	0.7477	0.8556
Mean	0.6990	0.8528	0.8566	0.8486	0.8540	0.7439	0.8530

**FIGURE 6:**
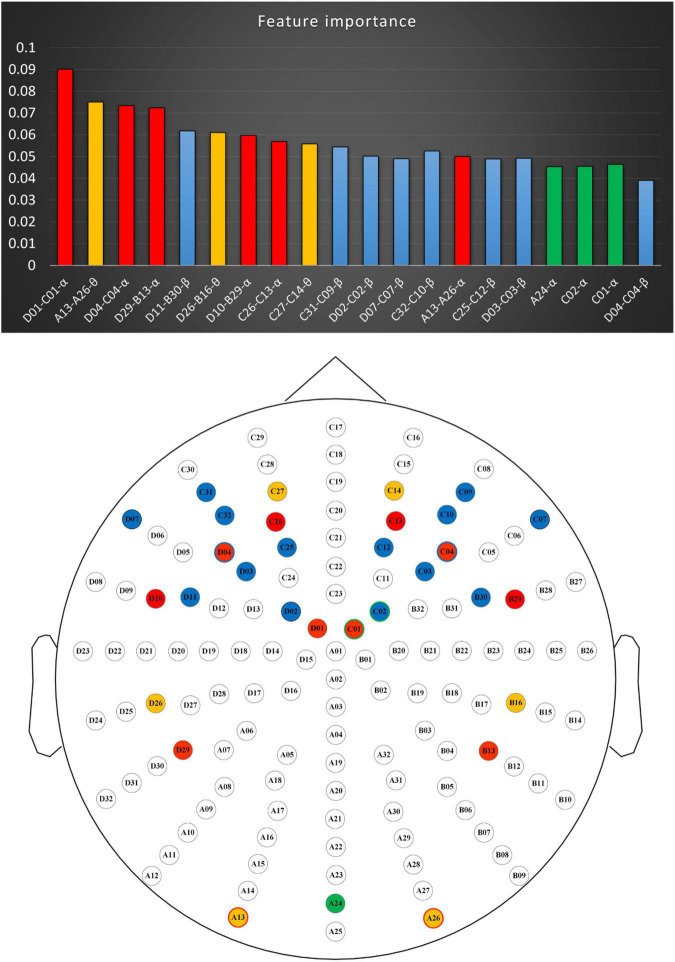
Optimal feature subsets of the RFC. Red, blue, and orange present the asymmetry features of the alpha, beta, and theta bands, respectively. Green represents the alpha band PSF. RFC, random forest classifier.

Figure 6 shows the union of the optimal feature subsets selected by each inner loop and the distribution of each feature. Based on the results shown in Figure 6, we found that the asymmetric features over the frontal and parietal lobes play an extremely important role in recognizing initial romantic interest because 15 of the 20 most discriminating features originated from these two regions. Moreover, 14 of these 15 features belonged to the alpha and beta bands. Previous studies have demonstrated that the frontal and parietal lobes are the most informative regions of emotional states, while the alpha and beta waves appear to be the most discriminative (Alarcao and Fonseca, 2019; Zheng et al., 2020). Yuan and Liu (2021) found that the changes in alpha and beta power on the sensors over the anterior regions play an important role in the generation and evaluation of IRA. In addition, numerous studies have demonstrated that frontal and parietal asymmetries in the alpha and beta FBs are observable for valence and arousal recognition (Cacioppo, 2004; Huang et al., 2012; Alarcao and Fonseca, 2019). In particular, Aldayel et al. (2020a,b) showed that the asymmetric features in alpha and beta frequencies over the frontal and parietal regions can effectively identify users’ emotional responses and preferences to market stimuli (Touchette and Lee, 2016; Liu et al., 2018; Ramsoy et al., 2018). In addition, Naser et al. showed that asymmetric features of alpha frequency on the frontal and parietal lobe regions could effectively identify users’ preference for music (Naser and Saha, 2021). We also found that the asymmetric features in the alpha, beta, and theta bands over the lateral occipital complex and the asymmetric features in the theta bands over the frontal and parietotemporal regions were sensitive in recognizing IRA. Previous studies have observed that the generation of IRA leads to desynchronization of alpha and beta bands in the lateral occipital complex region (Yuan and Liu, 2021). The theta FB over the frontal and parietotemporal areas was also considered to be an effective feature for identifying emotional states (Aftanas et al., 2001; Cartier et al., 2012).

Taken together, these findings demonstrate that users’ preferences for potential romantic partners can be determined on the basis of EEG signals. Furthermore, the TF features from channels over the frontal, parietal, and occipital regions are informative and suitable for the identification of IRA toward potential partners.

## Conclusion

The purpose of this experiment was to determine the possibility of using EEG signals to identify users’ aesthetic preferences for potential romantic partners. In this study, our system achieved a best accuracy of 85.2% (SD = 0.03) in recognizing the initial romantic interest. This result demonstrated that based on the information provided by users’ EEG signals, we can determine whether they are romantically interested in a potential partner. In addition, the best accuracy 85.2% (SD = 0.03) in this study was obtained mainly using the asymmetry features of the alpha, beta, and theta FBs on the sensors over the frontal lobe, parietal lobe, and lateral occipital complex. These results suggest that the TF features from channels over the frontal, parietal, and occipital regions are suitable for identifying human preferences for potential romantic partners. Therefore, in future work, we plan to extract features from different dimensions, such as the time domain and source domain, and explore how to use the minimum channels to optimize the classification accuracy through multi-dimensional feature integration.

In addition, as an exploratory study, this study used portrait photos rather than real people as stimuli to induce IRA based on feasibility considerations. The advantage of this approach is that by increasing the amount of stimulus, it can effectively solve the problem of insufficient trials in which IRA was successfully induced due to the low average induction rate. However, in real social scenes, initial romantic interest usually occurs in the environment that allows some meaningful interaction, but the types of stimuli used in the present study did not allow participants to interact effectively with potential partners in the photos (Yuan and Liu, 2021; Yuan et al., 2021). This is a problem that needs to be paid attention to and solved in the follow-up research. It is believed that in the near future, mate preference recognition and matching systems based on EEG signals will be applied to online or offline dating scenarios to assist individuals in finding their ideal partners.

## Data Availability Statement

The raw data supporting the conclusions of this article will be made available by the authors, without undue reservation.

## Ethics Statement

The studies involving human participants were reviewed and approved by Ethical Review Committee of Southwest University. The patients/participants provided their written informed consent to participate in this study. Written informed consent was obtained from the individual(s) for the publication of any potentially identifiable images or data included in this article.

## Author Contributions

GY and GL conceived the study. GY designed and programmed the tasks, collected the data, analyzed the composite behavioral and EEG data, and wrote the manuscript. GY and WH interpreted the results. GL and WH revised the manuscript. All authors approved the final manuscript.

## Conflict of Interest

The authors declare that the research was conducted in the absence of any commercial or financial relationships that could be construed as a potential conflict of interest.

## Publisher’s Note

All claims expressed in this article are solely those of the authors and do not necessarily represent those of their affiliated organizations, or those of the publisher, the editors and the reviewers. Any product that may be evaluated in this article, or claim that may be made by its manufacturer, is not guaranteed or endorsed by the publisher.

## Footnotes

1
http://bbs.fengniao.com/
